# Targeting BPTF Sensitizes Pancreatic Ductal Adenocarcinoma to Chemotherapy by Repressing ABC-Transporters and Impairing Multidrug Resistance (MDR)

**DOI:** 10.3390/cancers14061518

**Published:** 2022-03-16

**Authors:** Raúl Muñoz Velasco, Paula Jiménez Sánchez, Ana García García, Raquel Blanco Martinez-Illescas, Ángela Pastor Senovilla, Marian Lozano Yagüe, Alfonsina Trento, Rosa María García-Martin, Diego Navarro, Bruno Sainz, José Luis Rodríguez Peralto, Víctor Javier Sánchez-Arévalo Lobo

**Affiliations:** 1 Molecular Oncology Group, Biosanitary Research Institute, Faculty of Experimental Sciences, Francisco de Vitoria University (UFV), 28223 Madrid, Spain; raul.munoz@ufv.es (R.M.V.); paula.jimenez@ufv.es (P.J.S.); ana.garcia@ufv.es (A.G.G.); raquelbmimr@gmail.com (R.B.M.-I.); angela.cuellar98@gmail.com (Á.P.S.); marianly187@gmail.com (M.L.Y.); 2 Pathology Department, Hospital 12 de Octubre, Av. Córdoba, s/n, 28041 Madrid, Spain; altrento@yahoo.com (A.T.); rgarciamartin4@salud.madrid.org (R.M.G.-M.); jrperalto@salud.madrid.org (J.L.R.P.); 3 Department of Cancer Biology, Instituto de Investigaciones Biomédicas Alberto Sols (IIBM), CSIC-UAM, 28029 Madrid, Spain; dnavarro@iib.uam.es (D.N.); bsainz@iib.uam.es (B.S.J.); 4 Chronic Diseases and Cancer Area 3-Instituto Ramón y Cajal de Investigación Sanitaria (IRYCIS), 28029 Madrid, Spain; 5 Centro de Investigación Biomédica en Red, Área Cáncer, CIBERONC, ISCIII, 28029 Madrid, Spain

**Keywords:** BPTF, pancreatic cancer, ABC-transporters, multidrug resistance, gemcitabine

## Abstract

**Simple Summary:**

Pancreatic ductal adenocarcinoma is a devastating disease and an extremely chemoresistant tumour. In the present manuscript, we described the role of BPTF during tumour pancreatic ductal adenocarcinoma progression and in response to gemcitabine treatment, a gold standard treatment in this tumour type. Through different genetic approaches, we reduced BPTF levels in a panel of pancreatic ductal adenocarcinoma cell lines. We validated its therapeutic effect in cell cultures and in mouse models of pancreatic cancer. A reduction in BPTF levels impaired cell proliferation and sensitized pancreatic tumour cells to gemcitabine. We demonstrated that BPTF-silencing reduced the expression of several ABC-transporters, which are involved in gemcitabine resistance, and enhanced its accumulation in the tumour cell, improving its therapeutic effect.

**Abstract:**

Pancreatic ductal adenocarcinoma (PDA) is characterized by an extremely poor prognosis due to its late diagnosis and strong chemoresistance to the current treatments. Therefore, finding new therapeutic targets is an urgent need nowadays. In this study, we report the role of the chromatin remodeler BPTF (Bromodomain PHD Finger Transcription Factor) as a therapeutic target in PDA. BPTF-silencing dramatically reduced cell proliferation and migration in vitro and in vivo in human and mouse PDA cell lines. Moreover, BPTF-silencing reduces the IC50 of gemcitabine in vitro and enhanced its therapeutic effect in vivo. Mechanistically, BPTF is required for c-MYC recruitment to the promoter of ABC-transporters and its downregulation facilitates gemcitabine accumulation in tumour cells, increases DNA damage, and a generates a strong synergistic effect in vivo. We show that BPTF is a therapeutic target in pancreatic ductal adenocarcinoma due to its strong effect on proliferation and in response to gemcitabine.

## 1. Introduction

Pancreas ductal adenocarcinoma (PDA) is a devastating disease with a survival rate of less than 10% [1]. This poor prognosis is due to the late diagnosis and the chemo resistance to available treatments. The only curative therapy is radical surgery. Up to 70% of tumours, initially classified as resectable, present surgical margins affected and a high rate of recurrence. Therefore, adjuvant chemotherapy is used for all resected pancreatic tumours without prior neoadjuvant therapy using gemcitabine-based protocols, gemcitabine with capecitabine or 5-Fluoruracil, or FOLFIRINOX (oxaliplatin, irinotecan with leucovorin, and infusion of short duration of 5-FU) [2]. Unfortunately, the disease progresses because of its high chemoresistance, both intrinsic and acquired, a phenomenon known as multidrug resistance (MDR), thus finding new therapeutic options is an urgent need [3,4].

The initial model used to explain the origin and progression of PDA was a linear model where sequential histological abnormalities—termed Pancreatic Intra epithelial Neoplasia (PanIN)—were linked to specific molecular events. The earliest precursor lesions, namely PanIN-1A and 1B, are characterized by the presence of *KRAS* mutations and PanIN 1B is characterized by the genetic inactivation of *CDKN2A*. As PanINs progresses to PanIN-2, they inactivate or lose the *SMAD4* gene. Finally, PanIN-3, considered as carcinoma in situ, is characterized by *TP53* losses [5]. Next-generation sequencing studies allowed for the identification of new mutations and the classification of PDA into subgroups with prognostic and predictive value [6,7,8,9,10]. For example, the combined FOLFIRINOX regimen increases survival in patients with molecular alterations in *BRAC1*, *BRAC2*, or *PALB2* present in the unstable subtype of the classification of Waddell et al. [10]. Mutations in *PI3KCA* and *EGFR* allow for the use of specific inhibitors but with limited benefits [11]. Thus, it is essential to identify new therapeutic targets and epigenetic regulators are promising targets to develop specific inhibitors [12]. For example, the BRD4 inhibitor JQ1, in which BRD4 is a member of the BET family (Bromodomain and Extra Terminal domain), has shown therapeutic potential in patient-derived xenografts by impairing tumour progression and sensitizing tumours to PARP inhibitors [13,14]. JQ1 synergizes with gemcitabine by enhancing DNA damage [14,15] and, in combination with Histone deacetylase inhibitors, delays tumour progression [16]. A new BET inhibitor, namely I-BET762, can inhibit PDA cell proliferation and synergizes with gemcitabine in vitro and in vivo [17]. These results support the importance of epigenetic regulators as therapeutic targets.

Among the chromatin remodelers, BPTF stands out as a member of the NURF complex that recognizes H3K4me3 and H4K16ac to promote nucleosome sliding and H1 exchange [18,19]. The recognition of those marks and the subsequent remodelling are required for c-MYC chromatin recruitment and its transcriptional activity [20]. c-MYC is amplified in PDA and regulates the expression of the ATP-binding cassette (ABC) transporters, which increases the drug efflux responsible for PDA chemoresistance [4,21,22,23]. BPTF copy number gains have been detected in several tumour types, such as lung, prostate, and neuroblastoma [24]. Our previous work showed the therapeutic potential of BPTF in a PDA mouse model driven by c-MYC (*Ela1-c-MYC*), reducing cell proliferation and tumour growth [20]. The main limitation of this study was the use of a PDA mouse model independent of *KRAS*, which is the main driver mutation in PDA. To overcome this issue, in this study, we aim to validate the therapeutic value of BPTF by itself and in combination with gemcitabine in PDA *KRAS*-driven tumours either in vitro or in vivo using preclinical immunodeficient and immunocompetent mouse models.

## 2. Materials and Methods

### 2.1. Cell Culture

Hek293T and human PDA cells, namely Patu 8988T, Patu 8988S, Panc1, and T3M4, were cultured in Dulbecco’s modified Eagle’s medium (DMEM; LONZA 12-604F) supplemented with 10% fetal bovine serum (FBS TICO EU (A3FBSEU500)) and penicillin/streptomycin (Life Technologies, Carlsbad, CA, USA). All these cell lines were kindly provided by Dr. Francisco X Real (Centro Nacional de Investigaciones Oncologicas, CNIO, Calle de Melchor Fernández Almagro, 3, 28029, Madrid, Spain)

### 2.2. shRNAs Lentiviral Vector Constructs and Virus Production

Mission shRNAs (Sigma-Aldrich, St. Louis, MO, USA) were used for RNA-interference. A BPTF-targeting shRNA (shBPTF-1, clone TRCN0000016819) was used and compared to a control non-targeting shRNA. shRNAs were kindly provided by Dr. Francisco X Real (CNIO). Lentiviral production: infectious lentiviruses were produced in Hek293T cells by Jet prime transfection of the lentiviral construct together with the packaging plasmids psPAX2 and pCMV-VSV-G. Post-transfection (48 h): The medium was harvested twice for an additional 24 h. Viral supernatants were filtered and either frozen down in aliquots or applied on target cells in the presence of 5 µg/mL polybrene. Cells were used after 72 h puromycin selection.

### 2.3. CRISPRi

The system includes the following plasmid: pLV hU6-sgRNA hUbC-dCas9-KRAB-T2a-Puro (VJ603), Addgene number 71236. The cloning of gRNAs was done using the restriction enzyme BsmBI as described in Thakore et al. [25]. gRNA sequences are provided in Supplementary Table S1. The gRNAs-design was performed by selecting the region +50 +200 bp from the TSS and using the websites CRISPOR [26] and Benchling (Biology WebSoftware; 2022; retrieved from https://benchling.com accessed on 6 April 2019). Virus production was performed as described in the previous section.

### 2.4. Growth and Colony Assays

To determine viability, 2 × 10^3^ cells per well were seeded in 96-well plates. After 1, 2, 3, 4, and 7 days, cells were fixed with 0.5% glutaraldehyde, washed twice with PBS 1×, and incubated with 0.5% crystal violet in 25% methanol; crystal violet was eluted with 10% acetic acid and the OD590 nm was determined. To assess colony formation, 5 × 10^2^ cells were seeded in 6-well plates and, after 14 days, cells were processed as described earlier.

### 2.5. Migration Assays

For transwell assays, 2 × 10^5^ cells were seeded in each upper chamber in 0.1% FBS DMEM and placed in a well with 10% FBS DMEM. Next-day chambers were fixed with 0.5% glutaraldehyde, washed twice with PBS1X, and incubated with 0.5% crystal violet in 25% methanol. Each chamber was photographed using optical microscopy and analyzed using ImageJ. For wound-healing assays, cells were grown until they reached confluence in 10% FBS DMEM and then the media was replaced by 0.1% FBS DMEM. A wound was performed using a tip to scratch the plate longitudinally while wound-healing was monitorized during 4, 6, and 24 h. Wound-healing was quantified using ImageJ software.

### 2.6. Cell Cycle Assay

The cell cycle profile was determined using FxCycle™ Violet Ready Flow™ Reagent (R37166, ThermoFisher Scientific, Waltham, MA, USA) by flow cytometry by adding two drops or the reagent, followed by incubation and analysis. Results were analyzed by FlowJo v10.

### 2.7. Immunofluorescence

Immunofluorescence was performed in cells fixed with 4% formaldehyde in PBS for 15 min at room temperature. Cells were blocked and permeabilized in 5% BSA with 0.3% Triton X-100 for 60 min at room temperature. Cover slides were subjected to immunofluorescence staining with H2A.X Ser139 (1:400, Phospho-Histone H2A.X #9718, Cell Signaling) in 1% BSA and 0.3% Triton X-100 overnight. Next-day cover slides were incubated with Alexa Fluor 594 goat anti rabbit IgG (Life Technologies, A11012) for 2 h and then incubated 15 min with DAPI (Panreac AppliChem, C/ Garraf 2, Polígono Pla de la Bruguera E-08211 Castellar del Vallès (Barcelona)). Cover slides were mounted with the ProLong Gold antifade reagent (P36934, ThermoFisher Scientific, Waltham, MA, USA). Immunofluorescence was performed using THUNDER Imager (Leica, Wetzlar, Germany) and analyzed using ImageJ.

### 2.8. Drug Synergy Assays

Gemcitabine (ACCORD 691980.4 OH) IC50 was determined by a drug/response curve, 5 × 10^3^ cells were seeded into 96-well plates, and next-day different dilutions of Gemcitabine were used. After 72 h of treatment, cell viability was determined using ATP-Lite (Ref 6016731) using a luminometer reader (EnSpire^®^ Multimode Plate Reader).

### 2.9. Calcein-AM Assay

Cells (5 × 10^3^) were seeded in black 96-well plates. After 24 h, Calcein-AM uptake was determined using the Invitrogen Calcein-AM assay kit according to the manufacturer instructions (Life Technologies C1430). Fluorescence was determined at 494/520 nm using a luminometer reader (EnSpire^®^ Multimode Plate Reader, PerkinElmer, Inc. 940 Winter Street Waltham, MA, USA).

### 2.10. Quantitative Real-Time PCR

Total RNA was isolated from cultured cells using the NucleoSpin RNA (22740955.250) according to manufacturer’s instructions. Samples were treated with DNase I before reverse transcription. cDNA was generated from 1 μg RNA using random hexamers and reverse transcriptase (TaqMan Reverse Transcription Reagents N8080234). qPCR amplification and analysis were conducted using the 7500HT Real-Time PCR System (Applied Biosystems, ThermoFisher Scientific, Waltham, MA, USA) using the GoTaq(R) qPCR Master Mix (Promega, Madison, WI, USA). RNA levels were normalized to *HPRT* or *GAPDH* expression using the DDCt method. Primer sequences are provided in Supplementary Table S1.

### 2.11. In Vivo Xenograft Tumorigenic Assays

T3M4 and Patu 8988 T BPTF-silenced (gBPTF #6 and gBPTF #8), and their non-target counterpart (gSCR) cells were grown in 6 to 8-week-old female nude mice (Rj:ATHYM-Foxn1nu/nu, Janvier Laboratories, Le Genest-Saint-Isle, France). Cells (2 × 10^6^, 100 mL in PBS 40% matrigel) were subcutaneously implanted, growth was monitored using an electronic calliper, and volumes were calculated using the formula L × W^2^ × 0.5. Gemcitabine was administrated at a dose of 30 mg/kg every 2 days intraperitoneally; tumour volume was evaluated every 4 days. KPC cell lines BPTF-silenced and their non-target counterparts were subcutaneously implanted in C57/BL6 mice, and growth was monitored as described previously. For all in vivo experiments, mice were housed according to institutional guidelines and all experimental procedures were performed in compliance with the institutional guidelines for the welfare of experimental animals approved by the Hospital 12 de Octubre Ethics Committee (CEI 20/377) and La Comunidad de Madrid (PROEX 312.8/21), as well as in accordance with the guidelines for Ethical Conduct in the Care and Use of Animals as stated in The International Guiding Principles for Biomedical Research involving Animals, developed by the Council for International Organizations of Medical Sciences (CIOMS).

### 2.12. Chromatin Immunoprecipitation

Cells were fixed with 1% formaldehyde for 15 min at room temperature. Fixation was stopped by adding glycine (to 0.125 M) with an additional incubation of 5 min. Cells were collected by scraping, pelleted, and then lysed for 10 min in 1 mL of buffer LB1 (50 mM HEPES (pH 7.5), 140 mM NaCl, 1 mM EDTA, 0.5% NP-40, 0.25% Triton X-100, and 10% glycerol) supplemented with protease inhibitors (Qiagen, Valencia, CA, USA). After centrifugation at 3000× *g*, pelleted nuclei were resuspended in 1 mL of buffer LB2 (10 mM Tris (pH 8.0), 200 mM NaCl, 0.5 mM EGTA, and 1 mM EDTA) and incubated at room temperature for 10 min. Pelleted nuclei were resuspended in 1 mL of ChIP SDS buffer (100 mM NaCl, 50 mM Tris (pH 8), 5 mM EDTA pH 8, 0.2% NaN_3_, and 0.5% SDS) and sonicated for 10 min at 55% intensity in a Branson sonicator, yielding DNA fragments of 300–500 bp. Protein was quantified and 1.5 mg of protein was incubated with 10 μL of anti-MYC overnight at 4 degrees in a rotating platform. Beads were blocked overnight in PBS with 0.5% BSA and then added to the samples. After a 3 h incubation at 4 °C, beads were washed with Triton dilution buffer (100 mM Tris (pH 8.6), 100 mM NaCl, 5 mM EDTA (pH 8), 0.2% NaN_3_, and 5% Triton X-100), mixed micelle wash buffer (150 mM NaCl, 20 mM Tris (pH 8), 5 mM EDTA (pH 8), 5% sucrose, 0.2% NaN_3_, 1% Triton X-100, and 0.2% SDS), Buffer 500 (0.1% deoxycholic acid, 1 mM EDTA (pH 8), 50 mM HEPES (pH 7.5), 1% Triton X-100, 500 mM NaCl, and 0.2% NaN_3_), LiCl buffer (0.5% deoxycholic acid, 1 mM EDTA (pH 8), 250 mM LiCl, 0.5% NP-40, 10 mM Tris (pH 8), and 0.2% NaN_3_), and Tris-EDTA (TE). DNA was eluted in elution buffer and crosslinks were reversed by incubation overnight at 65 °C. RNA and protein were digested using RNase A and Proteinase K, and DNA was purified by both phenol–chloroform extraction and isopropanol precipitation. Target DNA abundance in ChIP eluates was assayed by qPCR with primer pairs designed to achieve products of 50–200 bp. Primer sequences are provided in the Supplementary Table. The following antibody was used: anti-MYC Cell Signaling c-Myc Antibody CST (1679402S).

### 2.13. Immunohistochemistry

Immunohistochemistry was performed on 4 µm thick sections of formalin-fixed, paraffin-embedded samples. Immunostaining was performed on a Leica Bond-III stainer (Leica Biosystem, Newcastle, UK). Ki67 staining was performed using a monoclonal mouse anti-human Ki67 antibody (clone MIB-1) that was ready to use (Dako Denmark A/S, Glostrup Kommune Denmark; BOND Polymer Refine Detection https://shop.leicabiosystems.com/en-es/ihc-ish/detection-systems/pid-bond-polymer-refine-detection accessed on 16 February 2022). Nuclei were counterstained with hematoxylin.

### 2.14. Bioinformatic Analysis

Expression values for *ABCC1*, *ABCC2*, *ABCC3*, and *ABCC4* in Eμ-Myc were downloaded from Gene Expression Omnibus series GSE141647. Expression and MYC-binding values for *ABCC1*, *ABCC2*, *ABCC3*, and *ABCC4* in WT and Eμ-Myc were downloaded from Sabò et al. [27]. Fold change in expression or binding was calculated relative to the control. Heatmaps were generated using the *seaborn* Python library.

### 2.15. Statistical Analysis

All quantitative data are presented as mean ± SD (standard deviation) from ≥3 different biological replicates. A comparison of the data that did not follow a normal distribution was performed using the Mann–Whitney test. Significance was considered for * *p* < 0.05, ** *p* < 0.01, *** *p* < 0.005, and **** *p* < 0.001. To compare the data distribution of two separate populations, we performed a two-way ANOVA. Software Prism 8.0 (GraphPad Software 2365 Northside Dr. Suite 560, San Diego, CA, USA) was used.

## 3. Results

### 3.1. BPTF Downregulation Impairs Tumour Progression in a Syngeneic KPC Mouse Model

**Figure 1:**
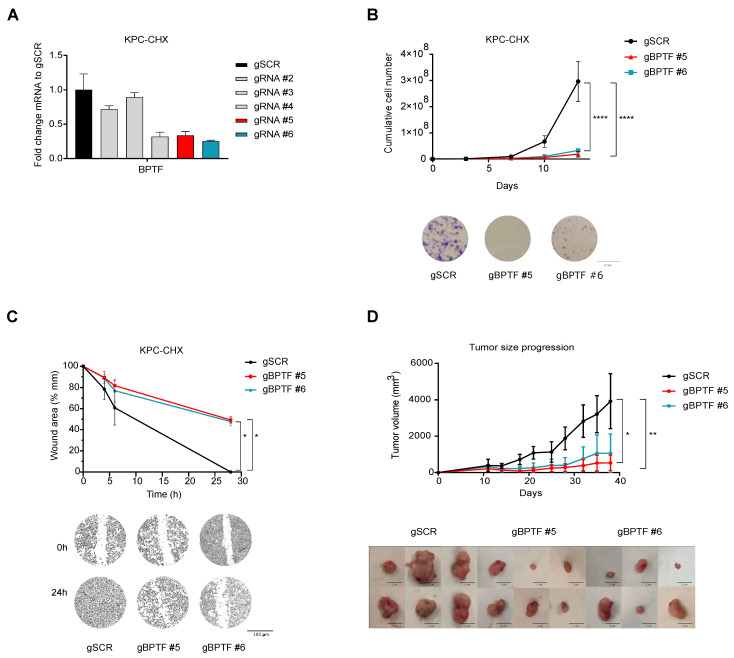
BPTF downregulation impairs tumour progression in a syngeneic KPC mouse model. (**A**) qRT-PCR for BPTF expression upon silencing with five specific gRNAs compared to non-targeted (gSCR) in KPC cell line; *n* = 3. (**B**) Proliferation and colony assay of BPTF-interfered (gBPTF #5 and gBPTF #6) cells compared to non-targeted (gSCR) in KPC cell line; *n* = 3. (**C**) Wound-healing assay of BPTF-interfered (gBPTF #5 and gBPTF #6) cells compared to non-targeted (gSCR) for 4, 6, and 24 h in KPC cell line; *n* = 3. (**D**) Tumour volumes in C57/BL6c mice subcutaneously implanted with BPTF-silenced (gBPTF #5 and gBPTF #6) KPC cells or non-targeted control cells (gSCR) by CRISPRi; tumours were measured for 37 days, *n* = 8. Representative images of the different tumours. Significance was considered for * *p* < 0.05, ** *p* < 0.01, and **** *p* < 0.001.

To assess the therapeutic value of BPTF inhibition during PDA progression, we used a KPC (LSL-Kras^G12D/+^; LSL-Trp53^R172H/+^; and Pdx1-Cre (KPC)-derived cell lines) syngeneic mouse model of pancreatic ductal adenocarcinoma (PDA) that recapitulates the human disease. We downregulated BPTF expression by CRISPRi to evaluate cell proliferation in vitro. We tested five different gRNAs targeting the region +20 +100 from the TSS and selected the best two for the following experiments (Figure 1A). To assess the role of BPTF in proliferation, we performed growth curves and colony assays after BPTF-silencing and observed a clear impairment in cell proliferation (Figure 1B). To evaluate the role of BPTF in migration, we performed a wound-healing assay; BPTF-silencing clearly impairs cell migration compared to its control counterpart (Figure 1C). To evaluate the role of BPTF during tumour progression in vivo, we subcutaneously implanted in C57/BL6 mice BPTF-silenced KPC cells or non-targeted control cells by CRISPRi and evaluated tumour growth. We observed that BPTF downregulation strongly reduces tumour progression in vivo (Figure 1D). Taken together, we can conclude that BPTF-silencing reduced cell proliferation and migration in vitro, as well as tumour growth in vivo.

### 3.2. BPTF Downregulation Impairs Cell Proliferation and Migration in a Panel of PDA Human Cell Lines

**Figure 2:**
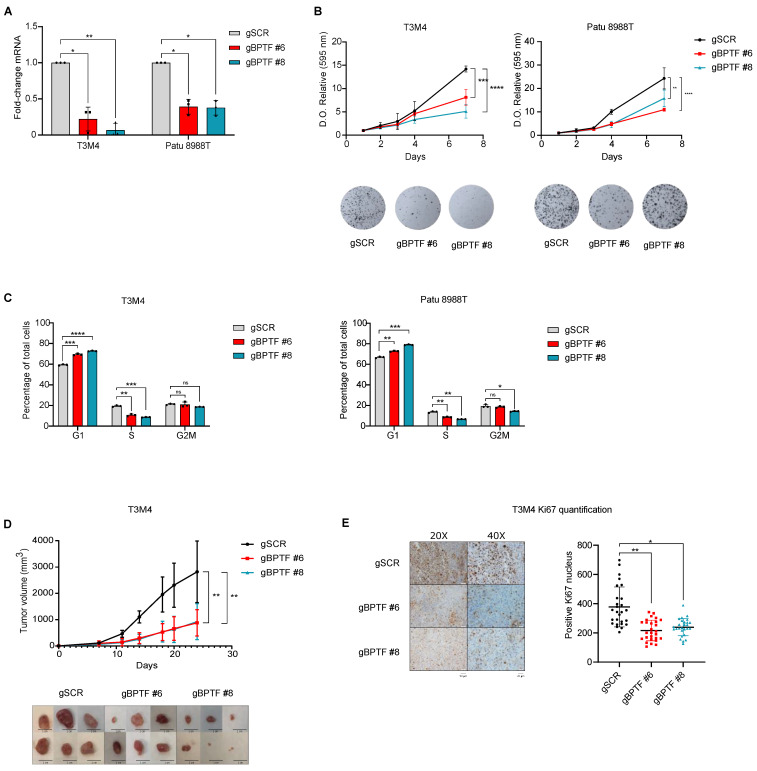
BPTF downregulation impairs tumour progression in human cell lines by CRISPRi. (**A**) Analysis of BPTF downregulation (gBPTF #6 and gBPTF #8) by RT-qPCR compared to non-targeted (gSCR) in T3M4 and Patu 8988T; *n* = 3 and schematic representation of gRNAs-design. (**B**) Proliferation and colony assay of BPTF-interfered cells compared to gSCR in T3M4 and Patu 8988T cell lines; *n* = 6. (**C**) Cell cycle assay by FACS of BPTF-interfered cells compared to gSCR in T3M4 and Patu 8988T cell line; *n* = 3. (**D**) Nude mice were subcutaneously implanted with either BPTF-interfered T3M4 cells (gBPTF #6 and gBPTF #8) or non-targeted (gSCR), and tumours were measured for 24 days; *n* = 8 and representative images of the different tumours. (**E**) Representative images of Ki67 immunohistochemistry and its quantification. Significance was considered for * *p* < 0.05, ** *p* < 0.01, *** *p* < 0.005, and **** *p* < 0.001, ns (not statistically significant).

To analyze if BPTF levels correlate with tumour proliferation, we assessed BPTF expression in a panel of 11 human PDA cell lines and selected four cell lines representative of the varying BPTF levels observed (Supplementary Figure S1A,B). PDA cells with high levels of BPTF proliferated faster (Supplementary Figure S1C). To demonstrate that BPTF is required for cells’ proliferation in human PDA tumours, we downregulated BPTF levels using CRISPRi. We designed specific gRNAs and selected the two best gRNAs (Figure 2A and Supplementary Figure S1D). We downregulated BPTF levels by CRISPRi in the aforementioned four human PDA cell lines—Patu 8988T, Patu 8988S, Panc1, and T3M4—and analyzed cell proliferation and colony growth (Figure 2A,B and Supplementary Figure S2A–C). These results were further validated using a specific shRNA (Supplementary Figure S3). As we expected, BPTF downregulation decreased cell proliferation in all the cell lines tested. This reduction was consistent with the accumulation of cells in the G1 phase of the cell cycle (Figure S2C). To confirm those results in vivo, we subcutaneously implanted in nude mice T3M4 and Patu 8988T silenced for BPTF or CRISPRi non-target controls. Consistent with our results using KPC-derived cells, we observed a dramatic reduction in tumour growth after BPTF-silencing in both human cell lines, along with reduced staining for the proliferative marker Ki67 (Figure 2D,E and Supplementary Figure S2D). With these results, we can conclude that BPTF is required for cell proliferation and tumour growth in human PDA.

**Figure 3:**
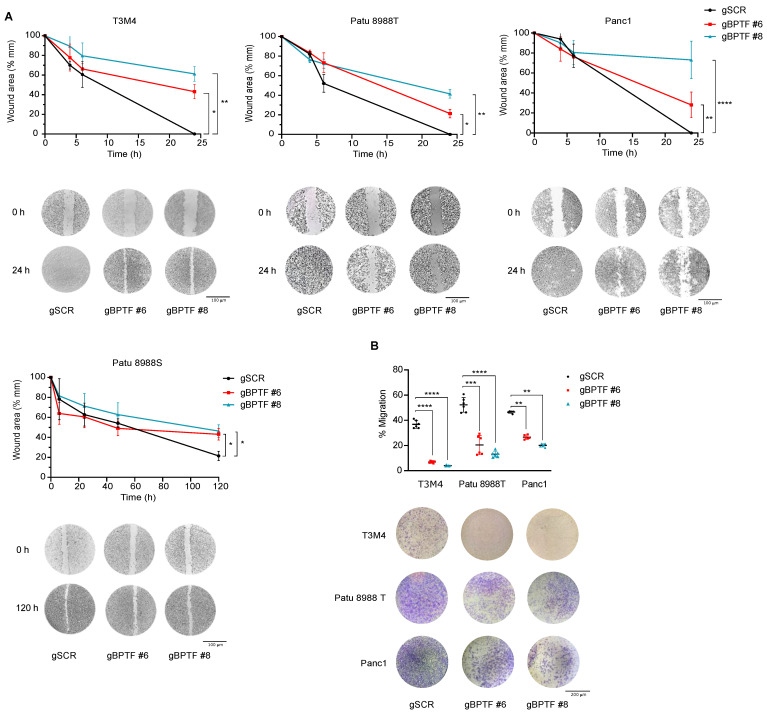
BPTF-silencing by CRISPRi impairs tumour migration in human PDA cell lines. (**A**) Wound-healing assay of BPTF-interfered cells (gBPTF #6 and gBPTF #8) compared to non-targeted (gSCR) for 4, 6, and 24 h in a panel of human PDA cell lines; *n* = 3. (**B**) Transwell assay of BPTF-interfered cells (gBPTF #6 and gBPTF #8) compared to non-targeted (gSCR) for 24 h in a panel of PDA cell lines; *n* = 6. Significance was considered for * *p* < 0.05, ** *p* < 0.01, *** *p* < 0.005, and **** *p* < 0.001.

To assess the role of BPTF during cell migration, we downregulated BPTF levels either by CRISPRi or shRNA, and evaluated migration in wound-healing and transwell assays. We observed that BPTF downregulation in Patu 8988T, Panc1, and T3M4 clearly impaired cell migration, showing a delayed wound closing or migration through the transwell (Figure 3 and Supplementary Figure S4). Patu 8988S, which expressed the lowest BPTF levels (Supplementary Figure S1B), did not migrate in transwell assays. Together, these results strongly support the role of BPTF in cell migration.

### 3.3. BPTF-Silencing Sensitizes to Gemcitabine

**Figure 4:**
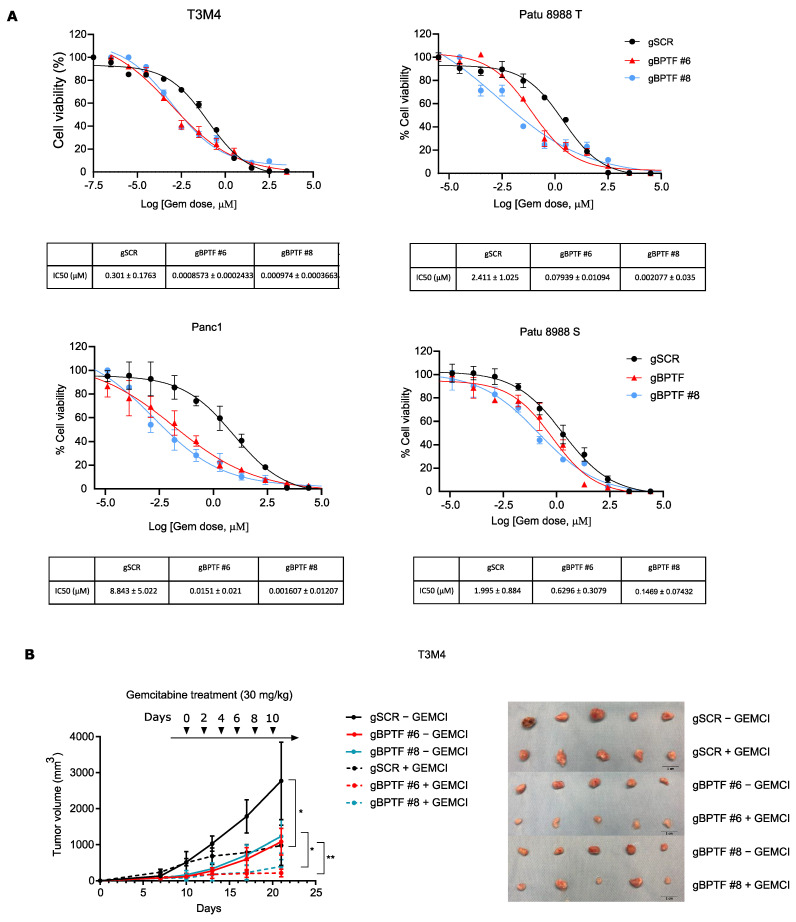
BPTF-silencing by CRISPRi sensitizes to gemcitabine. (**A**) IC50 analysis and values using ATP-lite in a panel of four PDA human cell lines either BPTF-interfered (gBPTF #6 and gBPTF #8) or non-targeted (gSCR); *n* = 3. (**B**) Tumour volumes of nude mice subcutaneously implanted with T3M4 cell lines either BPTF-interfered (gBPTF #6 and gBPTF #8) or non-targeted (gSCR); gemcitabine administration was done when tumour volume reached 200 mm^3^ at a dose of 30 mg/kg or PBS every two days; *n* = 8; and representative images of the different tumours. Significance was considered for * *p* < 0.05, ** *p* < 0.01.

To analyze if BPTF-silencing could synergize with gemcitabine treatment, we calculated the IC50 of gemcitabine for Patu 8988T, Patu 8988S, Panc1, and T3M4. In all the cell lines tested, BPTF-silencing either by CRISPRi or by shRNA dramatically reduced the IC50s (Figure 4A and Supplementary Figure S5). To validate this synergistic effect in vivo, we subcutaneously implanted in nude mice T3M4 BPTF-silenced and CRISPRi control cells and allowed the tumour to grow to 200 mm^3^. Afterwards, we administrated gemcitabine at a dose of 30 mg/kg or PBS every two days and monitored tumour growth. We observed a reduction in tumour growth after BPTF-silencing that was dramatically augmented with gemcitabine treatment (Figure 4B), indicating that BPTF-silencing indeed sensitizes tumour cells to gemcitabine. Thus, all these experiments clearly demonstrate for the first time that BPTF-silencing synergizes with gemcitabine in vitro and in vivo, leading to a strong tumour response.

### 3.4. The BPTF Regulates the Expression of the ABC-Transporters

**Figure 5:**
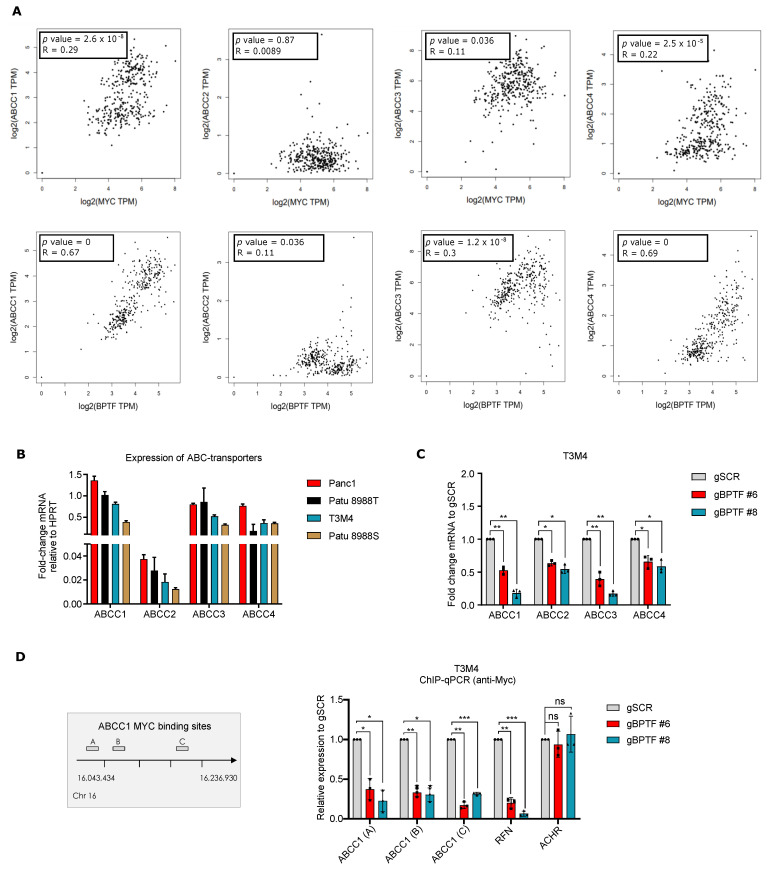
BPTF regulates the expression of several ABC-transporters. (**A**) Correlation between c-MYC, BPTF levels, and ABC-transporters’ expression using data obtained from Gene Expression Profiling Interactive Analysis (GEPIA). (**B**) qRT-PCR for ABCC1, ABCC2, ABCC3, and ABCC4 in a panel of four human PDA cell lines. (**C**) qRT-PCR for ABCC1, ABCC2, ABCC3, and ABCC4 of BPTF-interfered cells (gBPTF #6 and gBPTF #8) compared to non-targeted (gSCR); *n* = 3. (**D**) Chromatin immunoprecipitation of c-MYC on the ABCC1 promoter in BPTF-interfered cells (gBPTF #6 and gBPTF #8) compared to non-targeted (gSCR); *n* = 3. Significance was considered for * *p* < 0.05, ** *p* < 0.01, *** *p* < 0.005, ns (not statistically significant).

Different factors explain the limited response of PDA to gemcitabine and among them drug extrusion is an important mechanism of resistance. Several reports support the role of c-MYC as a direct regulator of ABC-transporters. Since BPTF is a c-MYC cofactor, we speculate that the c-MYC/BPTF axis might drive the expression of ABC-transporters and its inhibition enhances the gemcitabine response. To validate this hypothesis, we established a correlation between c-MYC, BPTF, and the expression of ABC-transporters using data obtained from the Gene Expression Profiling Interactive Analysis [28]. We unravelled a positive correlation between c-MYC, BPTF, and the transporters ABCC1, ABCC2, ABCC3, and ABCC4 (Figure 5A).

To validate this result, we established a correlation between BPTF levels and ABC-transporters in our panel of PDA cell lines. Supporting our previous results, there was a positive correlation between BPTF and the expression of specific transporters (Figure 5B). To confirm this correlation, we used T3M4 and downregulated BPTF levels by CRISPRi to analyze the expression of several transporters at the mRNA level, namely ABCC1, ABCC2, ABCC3, and ABCC4. There was a clear reduction in the expression of the evaluated ABC-transporters after BPTF-silencing (Figure 5C). Thus, these results demonstrate a strong correlation between BPTF, and the ABC-transporters required for the (MDR) phenotype. To demonstrate the role of BPTF and c-MYC in the transcription of the ABC-transporters, we used public available data from the *Eμ-Myc* mouse model, a classical model of lymphomagenesis to study c-MYC function [27]. We clearly observed c-MYC recruitment to the promoter of *ABCC1* and *ABCC4* in tumours from *Eμ-Myc* mice compared with controls that correlate with its higher gene expression (Supplementary Figure S6). Thus, to validate these results in a PDA cellular model and demonstrate that c-MYC requires BPTF to regulate its chromatin recruitment as well as the transcription of ABC-transporters, we analyzed c-MYC chromatin recruitment by ChIP-qPCR in T3M4 controls or BPTF-silenced on *ABCC1*. As a negative control, we used the acetylcholine receptor (*ACHR*). We observed a clear reduction in c-MYC recruitment in several regions previously described as c-MYC-binding sites [22] (Figure 5D). Thus, we can conclude that BPTF is required for c-MYC chromatin-loading in *ABCC1* and regulating its expression.

### 3.5. BPTF-Silencing Enhances Gemcitabine Accumulation and Increases DNA Damage

**Figure 6:**
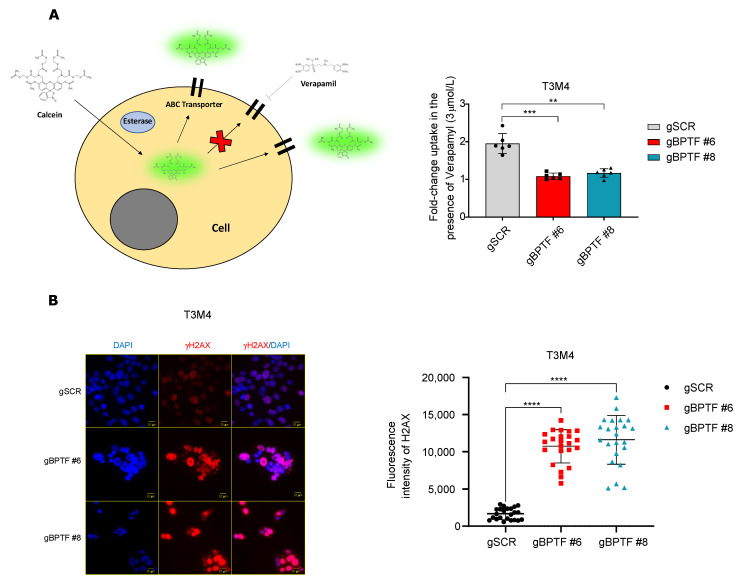
BPTF-silencing enhances gemcitabine action by increasing DNA damage. (**A**) Representative image of calcein-AM uptake as well as processing and export in the presence or absence of Verapamil. Fold change uptake of calcein in the presence of Verapamil (3 μmol/L) in T3M4 BPTF-interfered cells (gBPTF #6 and gBPTF #8) compared to non-targeted (gSCR); *n* = 3. (**B**) Representative images of immunofluorescence of **γ**H2AX staining and its quantification after 12 h of gemcitabine treatment (IC50) in T3M4 BPTF-interfered cells (gBPTF #6 and gBPTF #8) compared to non-targeted (gSCR); *n* = 6. Significance was considered for ** *p* < 0.01, *** *p* < 0.005, and **** *p* < 0.001, ns (not statistically significant).

**Figure 7:**
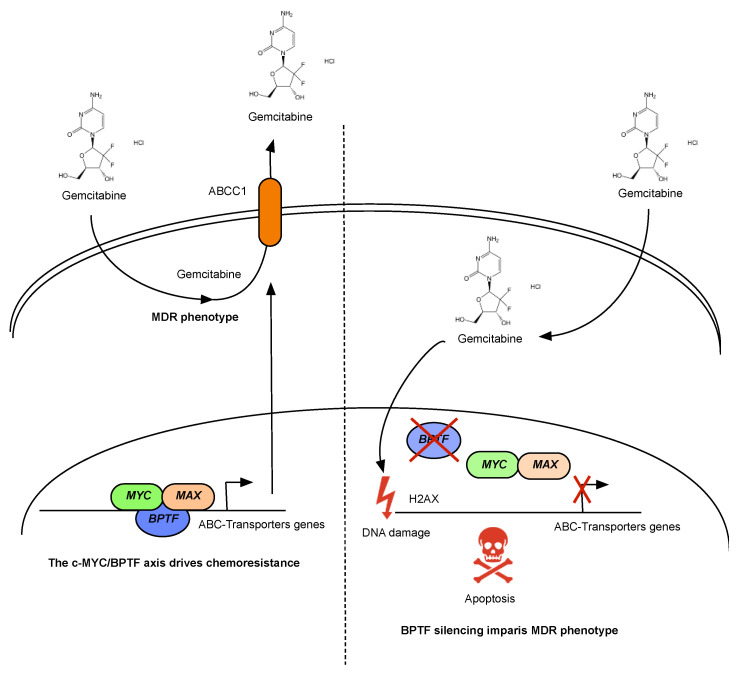
Schematic model of the synergism between BPTF-silencing and gemcitabine action. BPTF drives the expression of ABC-transporters in a c-MYC-dependent manner. BPTF inhibition impairs the c-MYC-dependent transcription of different ABC-transporters, avoiding the MDR phenotype and increasing DNA damage.

The aforementioned data strongly suggested that reduced ABC-transporter expression, due to BPTF-silencing, could impair the extrusion of gemcitabine and increase DNA damage. To demonstrate this hypothesis, we used, as a control, Verapamil, a specific inhibitor of P-glycoprotein and ABC-transporters [29,30], and calcein-AM. calcein-AM is a dye that passively enters in the cell through the membrane and is metabolized into calcein, a fluorescence molecule which can be extruded by ABC-transporters; however, this process is blocked by Verapamil [31]. As was expected, control cells treated with calcein-AM in the presence of verapamil showed a two-fold increased uptake of calcein, namely intracellular calcein, due to the inability of the cells to extrude the dye (Figure 6A). T3M4 BPTF-silenced cells showed a marked reduction in ABCC1, ABCC2, ABCC3, and ABCC4 transporters. Since there are less transporters to inhibit, BPTF-silenced cells became insensitive to verapamil and calcein was accumulated independently of the presence of verapamil (Figure 6A). The experiments with calcein and verapamil, along with the observed downregulation of ABC-transporters when BPTF was silenced, suggested the possible gemcitabine retention. Thus, we evaluated its effect at the level of DNA damage. We analyzed the levels of phospho gH2AX in the T3M4 control or BPTF-interfered cells in the presence of gemcitabine. We observed a higher degree of DNA damage due to gemcitabine in BPTF-interfered cells (Figure 6B), indicating that BPTF-interference impairs the expression of ABC-transporters, enhances gemcitabine accumulation, and increases the DNA damage response. These results mechanistically demonstrate the synergistic effect observed between BPTF-silencing and gemcitabine treatment (Figure 7).

## 4. Discussion

BPTF stands out as a member of the NURF complex that recognizes H3K4me3 and H4K16ac to promote nucleosome sliding and H1 exchange, and it is necessary for the transcriptional activity of the c-MYC oncogene [18,20]. We previously demonstrated that BPTF is required for c-MYC transcriptional activity, recruitment to low affinity binding sites, and chromatin remodelling [20]. We demonstrated the therapeutic value of BPTF in a mouse model of PDA driven by the c-MYC oncogene (*Ela1-Myc*) [20]; however, the main limitation of this study was the use of the pancreatic cancer mouse model, where tumorigenesis was driven by the *c-MYC* oncogene instead of *KRAS*, the main driver mutation in PDA [32]. To validate the therapeutic value of BPTF in cellular and mouse models driven by *KRAS* mutations, we used a panel of PDA cell lines and a KPC syngeneic mouse model [33,34]. In this model, the expression of mutant *Kras^G12D^* and *p53^R172H^* drives tumorigenesis to recapitulate the human disease [33]. In all models used, BPTF-silencing either by CRISPRi or shRNA reduced cell proliferation and tumour growth either in vitro or in vivo, supporting the results obtained in the *Ela1-Myc* mouse model.

Our data strongly demonstrate the therapeutic value of BPTF in response to gemcitabine through the regulation of ABC-transporters in a c-MYC-dependent manner. We demonstrated that BPTF-silencing impaired c-MYC recruitment to different binding sites on the *ABCC1* gene to regulate its transcription. Thus, upon BPTF-silencing, gemcitabine is accumulated in the cell, increasing DNA damage [32]. This result supports the data obtained by Zhao et al. who demonstrated that Verapamil, a specific inhibitor for MDR1 and MPR1 transporters, sensitizes PDA-resistant cells to gemcitabine by enhancing its accumulation [35] and strongly supports the role of BPTF as a therapeutic target. Different reports have highlighted the importance of c-MYC during the multidrug resistance phenotype in different tumour types. Kang et al. demonstrated how c-MYC amplification altered the expression of ABC-transporters in human breast epithelial cells [36] and Porro et al. showed how NMYC, another member of the *MYC* family, and c-MYC directly regulates its expression in different tumour types [22]. However, we cannot rule out that other transcription factors may participate in its regulation. A recent study by Wei et al. demonstrated that the TGFβ secreted by CAFs induces the expression of *ATF4* in tumour cells, a transcription factor that regulates the expression of *ABCC1* that enhances resistance to gemcitabine [37]. The relation between BPTF and ATF4 might be direct—through physical interaction—or indirect because BPTF can interact with SMADs in response to TGFβ driving *ATF4* expression [38]. Moreover, NFκB is another transcription factor associated with the expression of several ABC-transporters, especially in breast cancer [39,40], which might require BPTF for its transcriptional activity. Thus, future studies based on motifs enrichment analysis and chromatin immunoprecipitation will be required to clarify the contribution of the different transcription factors rather than c-MYC in PDA chemoresistance together with complementary studies with other chemotherapeutic agents to validate this synergistic effect and bypass its mechanism of resistance.

Several reports support the idea that ATP-dependent chromatin remodeler complexes are important therapeutic targets alone or in combination with gemcitabine [16,17] and potential novel treatment options are in progress [12]. The presence of specific domains in BPTF, such as the bromodomain (BRD) and two plant homeodomain finger (PHD) domains [41,42], could allow for the design of better specific inhibitors that might be transferred to the clinic in protocols of neoadjuvant chemotherapy alone or in combination with other agents. Xu et al. have shown in non-small cell lung cancer promising results with the specific BPTF inhibitor C620-0696 design to target the bromodomain [43] and Lu et al. have designed two new BPTF-inhibitors, namely DC-BPi-07 and DC-BPi-11, with therapeutic potential in MV-4-11 leukaemia cells [44]. Xiong et al. have reported two new BPTF inhibitors, namely Cpd8 and Cpd10, capable of downregulating c-MYC expression in A549 cell [44]. Thus, the development of these new inhibitors might help us to increase the therapeutic arsenal for this tumour type in future clinical trials either individually or in combination with gemcitabine.

## 5. Conclusions

In conclusion, this study supports the role of the BPTF as a therapeutic target in PDA. BPTF-silencing impairs cell proliferation and migration in PDA tumours, and strongly enhances the sensitivity to gemcitabine through the downregulation of ABC-transporters in a c-MYC-dependent manner.

## Data Availability

The data presented in this study are available on request from the corresponding author.

## Supplementary Materials

The following supporting information can be downloaded at: https://www.mdpi.com/article/10.3390/cancers14061518/s1, Figure S1: BPTF levels in PDA; Figure S2: BPTF downregulation impairs tumour progression in human cell lines by CRISPRi; Figure S3: BPTF downregulation impairs tumour progression in human cell lines by shRNA; Figure S4: BPTF impairs cells migration in a panel of human PDA by shRNA; Figure S5: BPTF-silencing by shRNA sensitizes to gemcitabine; Figure S6: Expression of ABC-transporters and c-MYC chromatin loading in data obtained from Eμ-Myc mice. Table S1: Primers and gRNA sequences.
