# Supplementary material for: Targeting BPTF Sensitizes Pancreatic Ductal Adenocarcinoma to Chemotherapy by Repressing ABC-Transporters and Impairing Multidrug Resistance (MDR)
Source: Cancers (Basel). 2022 Mar 16;14(6):1518. doi: 10.3390/cancers14061518 (PMC8946837; doi:10.3390/cancers14061518)
Supplement: Supplementary file 1 [file cancers-14-01518-s001.zip › Supplementary Figures.pdf]

cancers
MDPI

Supplementary Figures

## FIGURE SUPPLEMENTARY 1

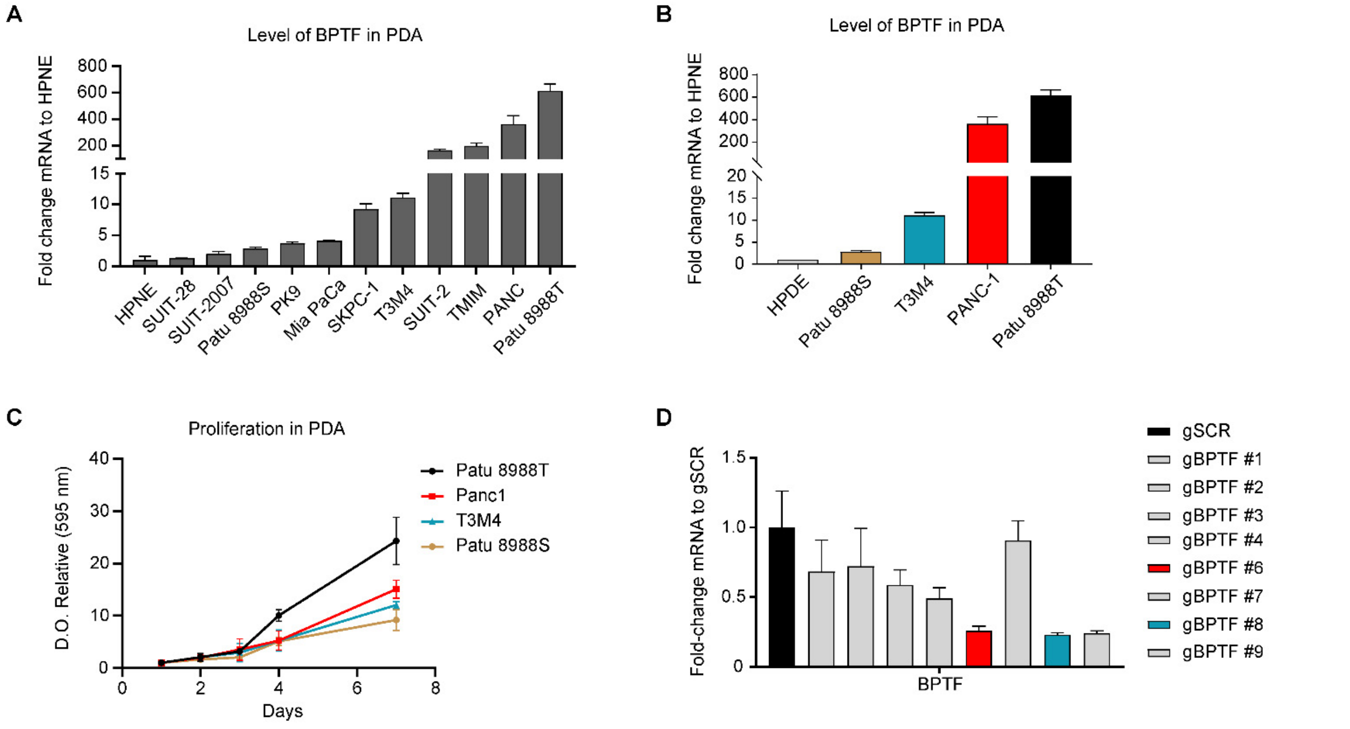


**Supplementary Figure S1.** BPTF levels in PDA. **(A, B)** qRT-PCR for BPTF in a panel of PDA cell lines compared to HPNE (Human Pancreatic Nestin Expressing cells), n = 3 **(C)** Proliferation assay of 4 PDA cell lines n = 3 **(D)** qRT-PCR for BPTF expression upon silencing with 9 different gRNAs against BPTF compared to non-targeted (gSCR), n = 3.

## SUPPLEMENTARY FIGURE 2

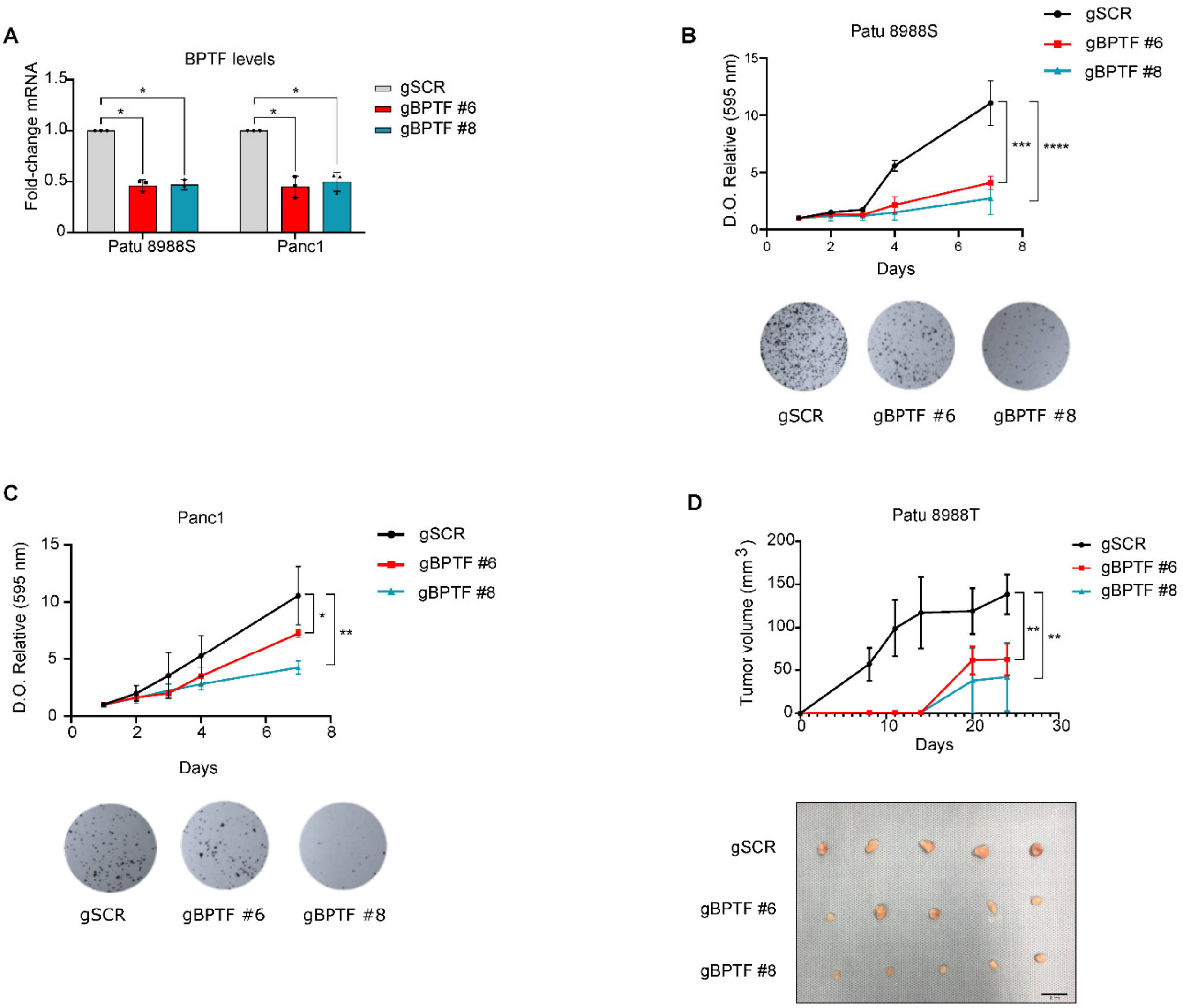


**Supplementary Figure S2.** BPTF downregulation impairs tumour progression in human cell lines by CRISPRi. **(A)** Analysis of BPTF downregulation (gBPTF #6 and gBPTF #8) by RT-qPCR compared to non-targeted (gSCR) in Patu 8988S and Panc1. n = 3. **(B)** Proliferation and colony assay of BPTF interfered cells compared to non-targeted (gSCR) in Patu 8988S cell line, n = 6. **(C)** Proliferation and colony assay of BPTF interfered cells compared to non-targeted (gSCR) in Panc1 cell line, n = 6. **(D)** Nude mice were subcutaneously implanted with either Patu 8988T cells BPTF-interfered (gBPTF #6 and gBPTF #8) or non-targeted (gSCR), tumors were measured for 24 days, n = 8.

## SUPPLEMENTARY FIGURE 3

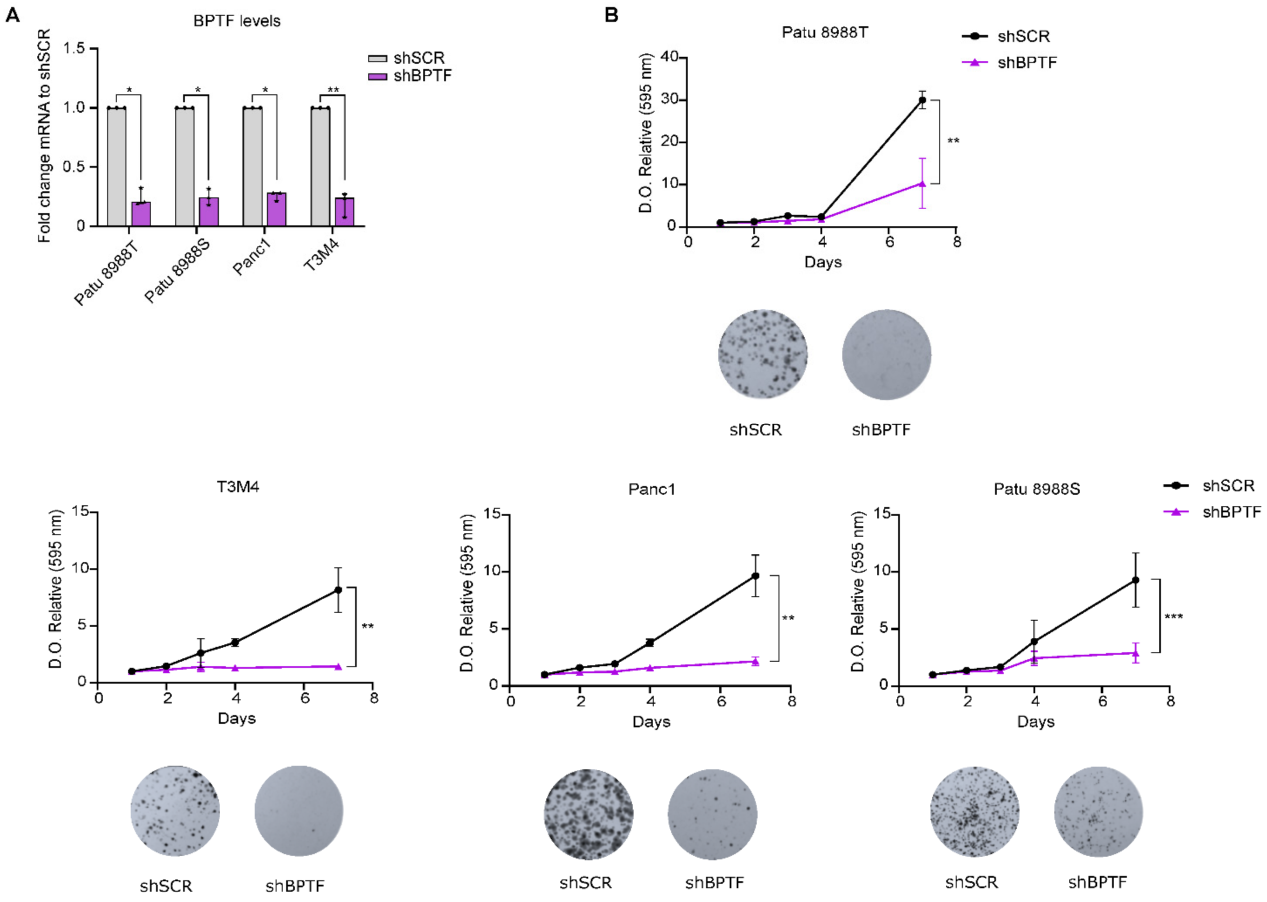


**Supplementary Figure S3**. BPTF downregulation impairs tumour progression in human cell lines by shRNA. **(A)** Analysis of BPTF downregulation using a specific shRNA, by qRT-PCR compared to non-targeted (shSCR) in a panel of 4 PDA cell lines, n = 3. **(B)** Proliferation and colony assay of shBPTF-interfered cells compared to shSCR in a panel of 4 PDA cell lines, n = 6.

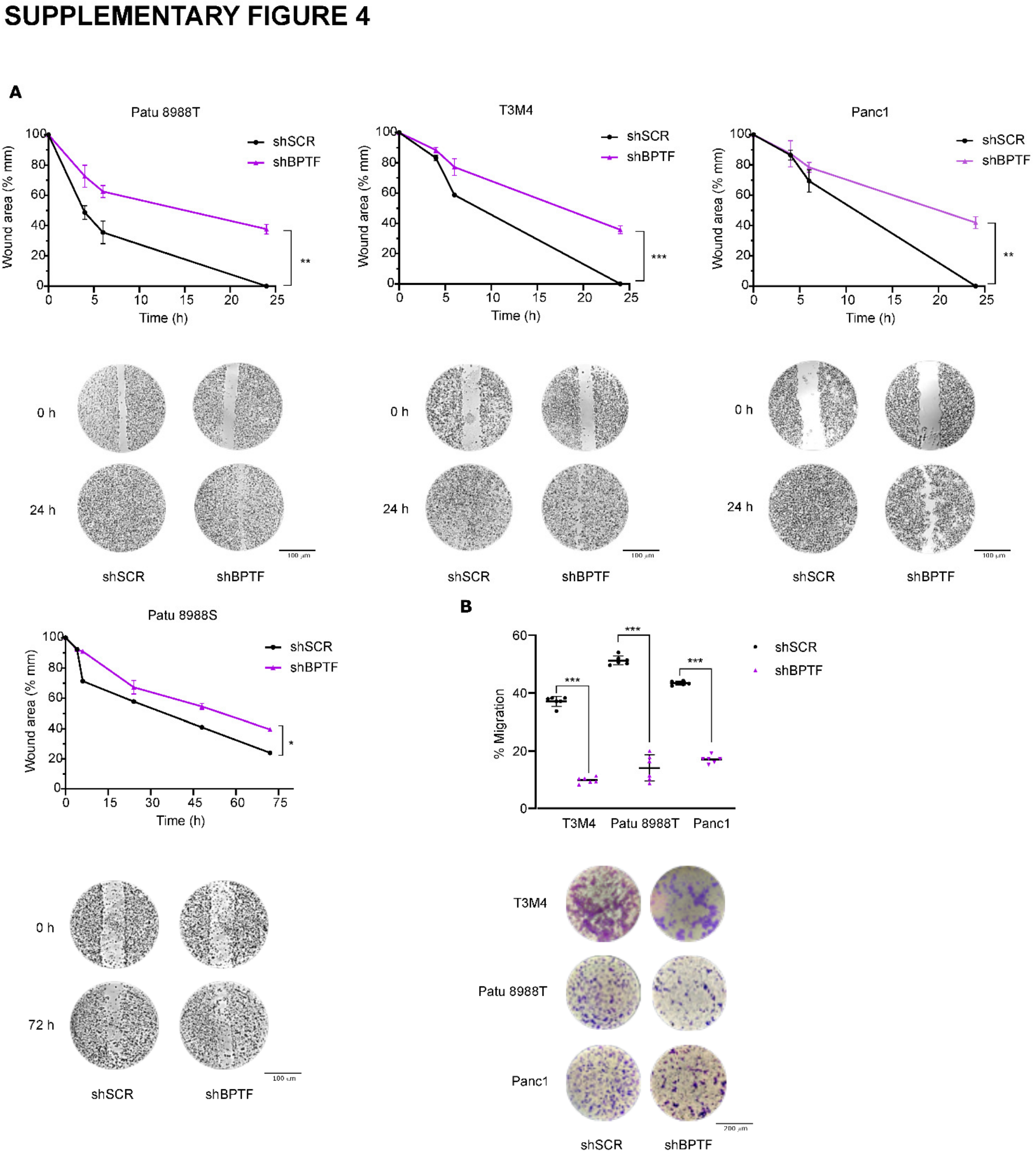


**Supplementary Figure S4**. BPTF impairs cells migration in a panel of human PDA by shRNA. **(A)** Wound healing assay of shBPTF-interfered cells compared to shSCR for 4, 6 and 24 h in a panel of human PDA cell lines, n = 3. **(B)** Transwell assay of shBPTF-interfered cells compared to shSCR for 24 h in a panel of human PDA cell lines, n = 6.

## SUPPLEMENTARY FIGURE 5

**A**

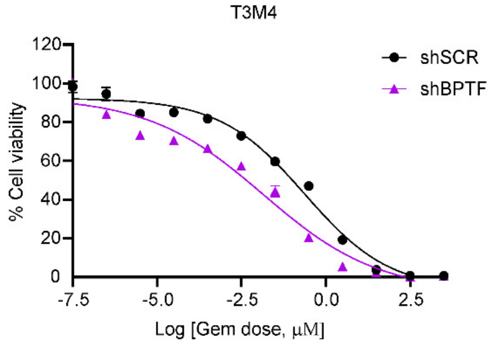


| | shSCR | shBPTF |
|---|---|---|
| IC50 (μM) | 0.2410 ± 0.133 | 0.01419 ± 0.009386 |

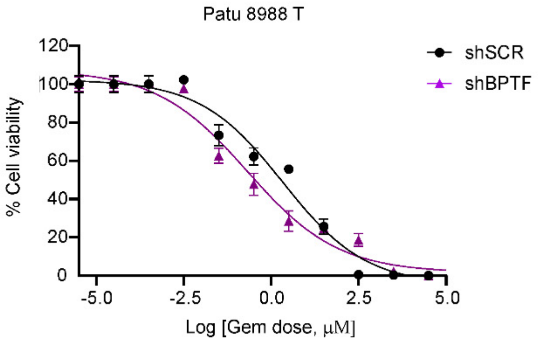


| | shSCR | shBPTF |
|---|---|---|
| IC50 (μM) | 2.166 ± 1.12 | 0.297 ± 0.1727 |

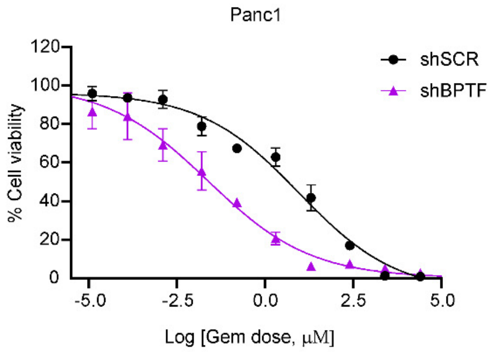


| | shSCR | shBPTF |
|---|---|---|
| IC50 (μM) | 9.031 ± 5.21 | 0.0241 ± 0.015 |

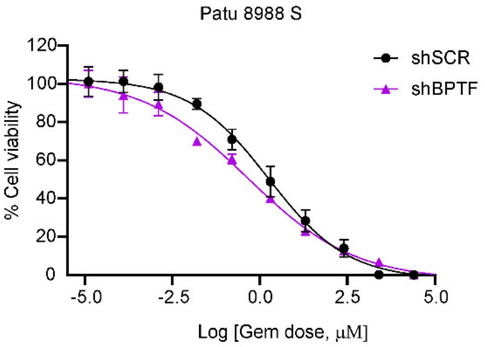


| | shSCR | shBPTF |
|---|---|---|
| IC50 (μM) | 1.811 ± 0.8 | 0.4593 ± 0.2087 |

**Supplementary Figure S5**. BPTF silencing by shRNA sensitizes to gemcitabine. **(A)** IC50 analysis and values using ATP-lite in a panel of 4 PDA cell lines either shBPTF interfered or non-targeted (gSCR), n = 3.

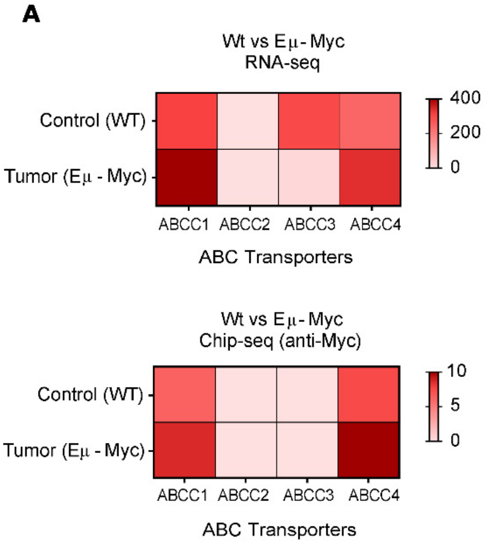


**Supplementary Figure S6**. Expression of ABC-transporters and c-MYC chromatin loading in data obtained from Eμ-Myc mice.
